# Perceptions of implementation of Massachusetts sports concussion regulations: results of a survey of athletic directors

**DOI:** 10.1186/s40621-020-00240-7

**Published:** 2020-04-20

**Authors:** Jonathan Howland, Julia Campbell, Linda Brown, Alcy Torres, Jonathan Olshaker, Richard Pearson, Courtney Hess

**Affiliations:** 1 Department of Emergency Medicine, Boston University Medical Center and Boston University School of Medicine, Boston, MA USA; 2 Injury Prevention Center, Boston Medical Center, Boston, MA USA; 3 Massachusetts Department of Public Health, Boston, MA USA; 4 Massachusetts Interscholastic Athletic Association, Franklin, MA USA; 5 Department of Counseling & School Psychology, University of Massachusetts Boston, Boston, MA USA

**Keywords:** Concussion, Mild traumatic brain injury, Athletic directors, Youth sports legislation

## Abstract

**Background:**

In 2011 the Massachusetts Department of Public Health issued regulations pursuant to 2010 Massachusetts youth sports concussion legislation that provided policies and procedures for persons engaged in the prevention, training, management, and return-to-activity for students who sustain head injury during interscholastic athletics, including Athletic Directors (ADs).

**Methods:**

A survey instrument was developed with participation from injury prevention experts at the Boston University School of Medicine, the Massachusetts Department of Public Health, and ADs. An electronic survey was sent to all AD members of the Massachusetts Interscholastic Athletic Association to assess their perceptions of implementation of the sports concussion law.

**Results:**

Response rate was 75% (260/346). The mean rating on a 0–10 scale (10 being “very important”) on importance of the law for student safety was 9.24, and the mean rating of the law’s impact on workload was 5.54. Perceived impact on workload varied as a function of whether or not the school also employed an athletic trainer (t = 2.24, *p* = 0.03). Most respondents (88%) reported that their school had a concussion management team, and 74% reported that they were informed “always” (31%) or “often” (43%) when a student-athlete experienced a head injury in a venue other than extracurricular sports. Most respondents (95%) endorsed that “all” or “most” school nurses were “very knowledgeable” about the law and regulations. Approximately half of all respondents endorsed that “all” or “most” teachers and guidance counselors were “very knowledgeable” about the law and regulations; 76% endorsed that “all” or “most” of students’ physicians were “very knowledgeable” about the law and regulations; 59% endorsed that “all” or “most” parents were “very knowledgeable” about the law and regulations. Sixty-six percent endorsed that student-athletes with concussion “often” (10%) or “sometimes” (56%) misrepresent their symptoms to accelerate return-to-play; and, 70% perceived that student-athletes with concussion “often” (15%) or “sometimes” (55%) misrepresent their symptoms to avoid academics.

**Conclusions:**

ADs perceive the sports concussion legislation as very important to student safety and positively assess implementation of the law and associated regulations. More effort is needed to increase understanding of the law among stakeholders including teachers, parents, and physicians.

## Introduction

Traumatic brain injury (TBI) is caused by a bump, blow, jolt, or penetrating injury to the head that disrupts brain function (Centers for Disease Control and Prevention 2019a). Concussion, or mild traumatic brain injury (mTBI), is common among children and adolescents and can result from impact to the head acquired during sports and recreational activity, vehicular crashes, assaults, or other injurious events. Annual incidence estimates of mTBI among youth ≤17 years range from 1 to 2 million (Bryan et al. 2016; Hardesty et al. 2019). It is estimated that between 2010 and 2016, over 2 million children and adolescents were treated for mTBI in US emergency departments (EDs) (Centers for Disease Control and Prevention 2019a; Sarmiento et al. 2019). Consequences of mTBI can present as cognitive impairments, including attention and memory deficits, physical impairments, such as compromised motor function, partial or complete loss of vision or hearing, headache, extreme fatigue or sleep dysregulation, and behavioral and emotional problems, such as diminished emotional regulation, depression, anxiety, aggression, and/or personality changes (Centers for Disease Control and Prevention 2019b).

The highest rates of ED visits for mTBI are among young males sustaining head impact while participating in contact sports, including football, basketball, hockey, lacrosse, wrestling, and soccer (Sarmiento et al. 2019). All 50 states have passed laws related to youth mTBI prevention and management. These laws tend to focus on mitigation of concussion through education of stakeholders, including students, parents, and school sports and teaching personnel, real-time diagnosis of concussion and removal from play, and medical clearance for return-to-activity (Lowery and Morain 2014). Most of these laws focus on head injuries occurring during school sports, but several include mTBI among all students, regardless of cause (Thompson et al. 2016).

In 2010, Massachusetts passed *Chapter 166, An Act Relative to Safety Regulations for School Athletic Programs*. A year later, the Massachusetts Department of Public Health (MDPH) issued accompanying regulations which stipulated policies and procedures for persons engaged in the prevention, training, and return-to-school management for students who sustain head injury during interscholastic athletics (Howland et al. 2018). These regulations target students in grades 6–12 and apply to all public and private middle and high schools that participate in the Massachusetts Interscholastic Athletic Association (MIAA). The regulations delineate the roles and responsibilities of School Nurses (SNs), Athletic Trainers (ATs), Athletic Directors (ADs), medical providers, students, and parents in responding to student head injury resulting from participation in extracurricular sports. Mandated responsibilities include annual trainings, using standardized forms for pre-sports participation, documenting and reporting head injury, developing a plan for return-to-school and ensuring medical clearance for return-to-play (Howland et al. 2018).

Proper implementation of laws and regulations is essential to the effectiveness of legislation (Lowery and Morain 2014). Accordingly, the MDPH has conducted or sponsored a series of studies to evaluate the implementation of the sports concussion regulations including adding questions to the Massachusetts Youth Risk Behavior Surveys and the Massachusetts Youth Health Surveys (Massachusetts Department of Public Health 2017) to estimate the incidence of mTBI among student athletes, and to assess response by ADs, ATs, and coaches. The MDPH has also conducted an analysis of school policies on sports concussion prevention and management (Brown et al. 2015), and has assessed the quality of mandated school reporting of student head injuries (Brown et al. 2015). In addition, the MDPH engaged the Injury Prevention Center (IPC) at Boston Medical Center (BMC) to conduct focus groups with SNs (Howland et al. 2018), ATs (Howland et al. 2018), and ADs, and to survey ADs about the implementation of the sports concussion regulations. These assessments aimed to provide MDPH staff with insight into how the law and regulations affected mTBI management in schools and problem areas in the implementation of the new legislation.

Results of the focus groups suggested that SNs, ATs, and ADs supported the law and felt supported by school administrators in implementing the regulations. SNs and ATs felt the law empowered them by providing more authority over students’ care and return-to-activity procedures than they had prior to implementation of the law (Howland et al. 2018). SNs, ATs, (Howland et al. 2018) and ADs felt that school teachers, counselors, athletic coaches, and healthcare providers required more education and training regarding sports concussion management. Interestingly, a subsequent survey of SNs conducted independently by the IPC confirmed a focus group finding that almost all SNs had generalized aspects of the sports concussion regulations to all students, regardless of how or where their head injury occurred (Hackman et al. 2018).

AD’s are responsible for overseeing all athletic programs at a school. The duties of ADs include hiring and supervising staff and coaches; managing team events, operations, scheduling, and finances; and keeping track of policy changes implemented by school boards, the MIAA, or the state. The Massachusetts sports concussion legislation requires ADs to conduct several mTBI trainings throughout the year and ensure compliance with sports-related regulations by parents, school staff, coaches, and student athletes. ADs are therefore significant stakeholders in the identification and management of mTBI among student athletes, and thus their viewpoint is important to a full understanding of the implementation of sports concussion regulations. Accordingly, the MDPH engaged the IPC to conduct a survey of ADs to assess their experiences implementing the sports concussion regulations. The results of survey are reported herein.

## Methods

### Survey instrument

#### Questionnaire development

The survey instrument was developed through an iterative process with input from IPC staff, MDPH staff, the Associate Director of Massachusetts Interscholastic Athletic Association (MIAA), and clinicians and researchers at the Boston University School of Medicine. Questions were developed in the following categories: (1) characteristics of schools and respondents; (2) salience of the sports concussion law and regulations; (3) diagnosis and management of mTBI; (4) workload impact of law and regulations; (5) assessment of stakeholder knowledge of law; and, (6) the extent to which students with mTBI misrepresent their symptoms during recovery (see survey questions in Additional file 1).

#### Characteristics of schools and respondents

Respondents were asked: (1) whether they were a member of the MIAA (response options: *yes*, *no,* or *don’t know*); (2) whether they were employed by a middle or high school (response options: *middle, high school*); (3) whether they were employed by a public or private school (response options: *public, private*); (4) whether their school employed an AT (response options: *yes*, *no,* or *don’t know)*; and, (5) the size range of their school's student population (response options: *up to 250, 251–500, 501–750, 751–1000, more than 1000*).

#### Salience of the sports concussion law and regulations

Respondents were asked to rate the importance of the sports concussion law and regulations to student health and safety using an 11-point Likert-type scale ranging from 0 (*not important*) to 10 (*very important*).

#### Diagnosis and management of return-to-activity

Respondents were asked: (1) whether their school had a concussion management (CMT) team for coordinating return-to-activity (response options: *yes*, *no,* or *don’t know)*; (2) the timeliness with which they were informed when a student athlete acquired mTBI in a venue other than interscholastic sports (response options: *always, often, sometimes, rarely*, or *never*); and, (3) whether, and for which students, their school provided baseline neuropsychological testing (BNT) (response options: *none of the students*; *all students engaged in certain extracurricular athletics at the schoo*l; *all students engaged in any extracurricular athletics at the school; all students in certain grades*; and, *all students in the school*).

#### Impact on workload

Respondents were asked to rate the impact of the sports concussion law and regulations on their workload using an 11-point Likert-type scale ranging from 0 (*not at all*) to 10 (*made it impossible to keep up with job demands*).

#### Assessment of stakeholders’ knowledge of the sports concussion law and regulations

Respondents were asked to estimate the proportion of various stakeholder groups who were knowledgeable about the sports concussion law and regulations (response options: *few, some, most, all* or *don’t know*).

#### Students misrepresenting concussion symptoms

Respondents were asked to estimate the frequency with which students with mTBI misrepresented their symptoms to: (1) return-to-play prematurely and (2) avoid academic tasks (response options for both questions: *always, often, sometimes, rarely*, or *never*).

#### Open-ended comments

Respondents were provided an opportunity to make comments or recommendations that they wished to convey to investigators (data not reported).

### Survey administration

The survey was conducted using Qualtrics survey software, licensed to BMC. All ADs who are employed at schools that are members of the MIAA received an email from the Associate Director of the MIAA that introduced the survey and contained a link to the questionnaire. The first survey mailing was sent in November 2018; non-respondents were sent a follow-up mailing in April 2019. The Qualtrics program prohibited more than one response from the same computer.

### Data analyses

#### Univariate analyses

Response frequencies to survey questions were compiled by Qualtrics and entered into data management files for analyses.

#### Bivariate analyses

We examined the associations between the school employing an AT and (1) school size and (2) whether the school was public or private. We examined the associations between a school having a CMT and (1) being public or private, (2) employing an AT, and (3) school size. We also examined the associations between provision of baseline neuropsychological testing and (1) school size and (2) whether the school was public or private. We examined whether rating of workload impact varied as a function of (1) whether schools employed an AT and (2) whether the school was public or private.

#### Multivariable analyses

We used linear regression to assess a model in which workload impact rating was the dependent variable and employed AT, school public vs. private, and school size were independent variables.

To assess differences in responses by categorical variables (e.g. school size ranges, public vs. private), we used chi-square tests. To assess differences in responses by cardinal variables (e.g., scale scores), we used t-tests. For regression analyses we used linear regression. Data analyses were performed using SAS version 9.4 and Microsoft Excel.

### Human subjects review

This study was reviewed and deemed exempt by the Institutional Review Boards at the Boston University Medical Campus and the MDPH.

## Results

### Response rate

There are 377 MIAA member schools. However, because some ADs serve more than one school, the MIAA schools employ a total 346 individual ADs. Of these, all were surveyed and 260 completed, or partially completed, the survey questionnaire. The overall response rate was 75% (260/346).

### School and respondent characteristics

All respondents (251/251) who answered this question indicated that they were a member of the MIAA. Ninety-nine percent (251/253) of respondents indicated that their responses were based on their employment at a high school; 83% (209/253) of respondents indicated that they were employed at a public (vs. private) school; and, 73% (184/251) of respondents indicated that their school employed an AT.

Private and public schools did not differ significantly with respect to the percentage that employed an AT (71% vs. 74%) or had a CMT (81% vs. 89%); larger schools, however, were significantly more likely than very small schools (up to 250 students) to have a concussion management team (*X*^*2*^ = 9.81; DF = 3; *p* = 0.02) (Table 1).

**Table 1:** Concussion management team by size of school

Concussion Management Team	School Size				
	≤ 250 (very small)	251–750 (small)	751–1000 (medium)	1000 + (large)	Total
No	8 (30%)	10 (9%)	6 (16%)	7 (9%)	31 (12%)
Yes	19 (70%)	97 (91%)	31 (84%)	73 (91%)	220 (88%)
Total	26	107	37	80	251

*X*^2^ = 9.81, df = 3, *p* = .02

### Salience of the sports concussion law and regulations

Respondents’ mean rating of the importance of sports concussion regulations to protect the health and safety of student athletes was 9.24 (SD: 1.39). Ninety-four percent (245/260) of all respondents answered this question.

### Diagnosis and management of mTBI

Eighty-eight percent (220/251) of respondents affirmed that their school had a CMT that meets to manage return-to-activity (learn and/or play) for students who have experienced mTBI or other brain injury. Seventy-four percent (182/245) of respondents believed that they were informed *always* or *most of the time* when a student athlete experienced mTBI or other brain injury that did not occur during extracurricular sports at their school. The majority of respondents (52% [124/238]) indicated that their school provided baseline neuropsychological testing for all of their students involved in extracurricular sports; 18% (43/238) of respondents indicated that their school did not provide baseline testing or comparable neuropsychological testing to any of their students; 13% (31/238) indicated that their school provided baseline testing for all students enrolled in certain extracurricular sports; 7% (16/238) indicated that their school provided baseline testing for all students; 6% (15/238) indicated that their school provided baseline testing for all students in certain grades, regardless of their extracurricular sports participation; and, 4% (9/238) indicated that their school provided baseline testing according to another formula (Table 2).

**Table 2:** Baseline neuropsychological testing practices

Student Baseline imPACT Testing	Count	Percent
No students	43	18
All students enrolled in extracurricular sports at the school	124	52
All students enrolled in certain extracurricular sports at the school	31	13
All students in certain grade levels	15	6
All students at the school	16	7
Other	9	4
Total	238	100

Private schools were more likely than public schools to provide baseline testing for all students, but not significantly so (13% vs. 5%; *p* = 0.09); private schools, however, were significantly more likely than public schools to offer baseline testing to none of their students (32% vs. 15%; *p* = 0.02) (Table 3). Medium and large schools were significantly more likely than small schools to provide baseline testing (*p* = 0.001) (Table 4).

**Table 3:** Student baseline testing by private vs. public school

Student Baseline imPACT Testing	School Type		
	Private	Public	Total
All	5 (14%)	11 (6%)	16 (7%)
Some	20 (54%)	150 (79%)	179 (75%)
None	12 (32%)	31 (15%)	44 (18%)
Total	37	191	238

*X*^2^ = 10.62, df = 2, *p* = .005

**Table 4:** Student baseline testing by school size

Student Baseline imPACT Testing	School Size			
	≤ 750	751–1000	1000 +	Total
All	5 (8%)	6 (6%)	5 (6%)	16 (7%)
Some	38 (58%)	78 (83%)	63 (81%)	179 (75%)
None	23 (35%)	10 (11%)	10 (13%)	43 (18%)
Total	66	94	78	238

*X*^2^ = 18.25, df = 4, *p* = .001

### Impact on workload

Respondents’ mean rating of the impact of implementing the sports concussion regulations on their workload was 5.54 out of 10 (SD = 2.35). Ninety-four percent (245/260) of all respondents answered this question.

The mean rating of the impact of regulations on workload was significantly lower (*p* = 0.03) among ADs at schools that employed an AT, compared to ADs at schools without ATs. The mean impact rating, however, did not vary significantly by school type (public vs. private), or school size. In a linear regression model with workload impact rating as the dependent variable and (1) AT employment, (2) school type (public vs. private) and (3) school size as independent variables, AT employment remained significantly associated with workload rating, controlling for school type and size (Table 5).

**Table 5:** Multivariable regression on workload impact rating

Impact on workload		
Variable	Beta (se)	***p***-value
AT employment	−0.86 (0.34)	0.01
Public school	0.57 (0.41)	0.17
School size	−0.04 (.16)	0.80

Model f-value = 2.77; *p*-value = 0.04; R^2^ = 0.0338

### Stakeholders’ knowledge of sports concussion regulations

Respondents estimated that 97% (238/245) of *all* or *most* of their peer ADs were knowledgeable about the sports concussion law and regulations. They estimated that 97% (236/244) of coaches, 95% (232/244) SNs, and 90% (214/237) of ATs were knowledgeable about the regulations. Respondents estimated lower levels of knowledge among other stakeholder groups. They estimated 76% (185/245) of *all* or *most* school administrators; 76% (185/244) of students’ physicians; 59% (144/243) of school guidance counselors; and, 59% (144/245) of students’ parents were knowledgeable about the regulations (Table 6).

**Table 6:** Proportion of stakeholders’ knowledgeable about sports concussion regulations

Stakeholder	All or most are knowledgeable		# Answering question (%)
	Count	Percent	
ADs	238	97	245 (94%)
Coaches	236	97	244 (94%)
School Nurses	232	95	244 (94%)
Athletic Trainers	214	90	237 (91%)
School Administrators	185	76	245 (94%)
Students’ physicians	185	76	244 (94%)
Guidance Counselors	144	59	243 (93%)
Students’ parents	144	59	245 (94%)
Teachers	102	42	245 (94%)

### Students misrepresenting concussion symptoms

Regarding return-to-play, 10% (24/234) of respondents indicated that students with mTBI *always* or *often* misrepresented their symptoms to accelerate re-engagement in extracurricular sports; 56% (131/234) indicated that students *sometimes* misrepresented their symptoms; and, 34% (79/234) indicated that students *rarely* or *never* misrepresented their symptoms in order to accelerate return-to-play.

Regarding avoidance of schoolwork, 14% (34/233) of respondents indicated that students with mTBI *always* or *often* misrepresented their symptoms to avoid schoolwork; 55% (128/233) indicated that students *sometimes* misrepresented their symptoms; and 31% (71/233) indicated that students *rarely* or *never* misrepresented their symptoms to avoid school work.

## Discussion

Our results indicate high support among ADs for the MA sports concussion law and regulations. This finding confirms the interpretation of the previous focus groups with ADs, and is consistent with the interpretations of the focus groups with SNs and ATs. The perceived impact the regulations have on daily workflow was slightly higher (5.5) for ADs than it was for SNs (5.0), when SNs were asked the same question (Hackman et al. 2018). It is likely that the impact of the regulations on workload for ADs is largely dependent on whether their school employs one or more AT. However, it is apparent, given the low R^2^ value in our regression model (R^2^ = 0.03), that there are other factors unmeasured by our survey that influence ADs perception regarding the impact of the regulation on their workload. The majority (87%) of respondents indicated that their school had a CMT, but further study is warranted to learn about the functioning of these teams relative to stakeholder membership and the frequency, methods, and quality of communication among team members.

Both the quantitative and qualitative data collected by this survey suggest issues in coordinating return-to-activity protocols with physicians. ADs gave physicians a moderate rating on knowledge of sports concussion regulations, relative to SNs, ATs, ADs, and coaches. This assessment of physicians’ understanding of, and compliance with, sports concussion regulations, confirms interpretations of the previous AD focus groups and of the focus groups with SNs and ATs (Howland et al. 2018). Other investigators have observed issues relative to the role of students’ healthcare providers in concussion diagnosis and management. Lowrey and Morain noted that sports concussion legislation in many states failed to specify the credentials and/or training requirements of healthcare providers who give medical clearance for return-to-activity (Lowery and Morain 2014). In a survey of MA primary care physicians, respondents reported limited communication with schools, and only 74% had taken a required clinical training course on concussions, despite a regulatory mandate to do so (Flaherty et al. 2016). In a qualitative study of MA student concussion management stakeholders, Doucette et al. noted the importance of cooperation from student athletes, their parents, and physicians for full implementation of the MA sports concussion legislation (Doucette et al. 2016). Short of legislating enforcement provisions to enhance physician participation in concussion management training, the MDPH might consider a collaborative program with the MA Medical Society and large provider groups to promote physician concussion training.

In our previous focus groups with SNs, ATs (Howland et al. 2018) and ADs, participants noted that some students misrepresent concussion symptoms in order to avoid academics or to accelerate return to athletics. Over half of the respondents to our survey endorsed that misrepresenting concussion symptoms occurs at least sometimes. Lowry and Morain noted that some parents “doctor shop” to obtain medical clearance for their children who have incurred a concussion playing extracurricular school sports (Lowery and Morain 2014). Such findings underscore the need for further education of parents and students relative to concussion risks.

The survey included several indicators of the quality of schools’ sports concussion programs: employing an AT, having a CMT, and the extent to which student baseline neuropsychological testing is provided by the school. Of these, two measures (employing an AT and having a concussion management team) did not differ significantly by whether the school was public or private, but all three measures differed significantly by school size. Further study is warranted to assess disparities in the cross-school quality of the diagnosis and management of sports concussion. School size may be a marker for the resources a school has to allocate to student health. It is important to assess whether variation in the the scope and quality of sports concussion management is associated with the economic status of the students.

Several limitations to our study are acknowledged. Participants may not have been representative of all MA ADs. It is possible that ADs that are employed at non-MIAA schools may have different experiences with, and perceptions of the law and regulations. Thus, generalizability to non-MIAA MA schools, and out-of-state schools should be approached with caution. Moreover, although the response rate was excellent for this type of study, there could be systematic differences in the schools that did not reply.

Thus far, our evaluations of implementation of the MA sports concussion law have involved focus groups with SNs, ATs, and ADs, and surveys of SNs and ADs (reported on herein). An additional survey of SNs is currently in the field. A complete picture of the implementation of the MA sports concussion legislation requires further studies that include students, parents, and students’ healthcare providers.

We did not perform psychometric evaluations on our survey instrument and therefore cannot attest to its validity or reliability. Nonetheless, development of the survey questions involved collaboration and input by experienced survey researchers, clinicians specializing in youth concussion, and ADs, which we believe, contributes to the valid interpretations of our questions.

## Conclusions

Massachusetts was among the early-adopter states in passing sports concussion legislation for student athletes, and among a minority of states that developed detailed regulations for implementing the law. This and other evaluations (Lowery and Morain 2014; Howland et al. 2018; Flaherty et al. 2016; Doucette et al. 2016) suggest that more effort is required to educate parents, teachers, guidance counselors, and healthcare providers about the regulations for management of post-mTBI return-to-activity. The MDPH has recently responded to this need by publishing and disseminating return-to-school guidelines (Massachusetts Department of Public Health 2018). This document aims to promote education and understanding among school staff, parents, students, and healthcare providers. Further evaluation will assess the distribution and impact of this initiative.

Various stakeholder organizations should be engaged in promoting awareness of the risks of youth concussion in general and the provisions of relevant legislation. Variation in the quality and scope of sports concussion legislation suggests the need for further enforcement powers for the public entities authorized to oversee these laws (Lowery &Morain, 2014). This study, and the other evaluations undertaken by the MDPH and other investigators, underscore the value of comprehensive continuous quality improvement for new and emerging public policy.

## Supplementary information

**Additional file 1.** Athletic Director’s Survey Questions

## Data Availability

The data that support the findings of this study are available from author, Julia Campbell, but restrictions apply to the availability of these data, which were used under license for the current study, and so are not publicly available. Data are however available from the authors upon reasonable request and with permission of the Massachusetts Department of Public Health.
