# Supplementary material for: Perceptions of implementation of Massachusetts sports concussion regulations: results of a survey of athletic directors
Source: Inj Epidemiol. 2020 Apr 20;7:13. doi: 10.1186/s40621-020-00240-7 (PMC7168948; doi:10.1186/s40621-020-00240-7)
Supplement: Supplementary file 1 — Additional file 1. Athletic Director’s Survey Questions [file 40621_2020_240_MOESM1_ESM.docx]

Athletic Director’s Survey Questions

Q1. Your answers are based on employment at a:

[ ] High school [ ] Middle school

Q2. Your school is:

[ ] Public [ ] Private

Q3. Approximately how many students are currently enrolled in your school?

[ ] Up to 250 [ ]251-500 [ ]501-750 [ ] 751-1,000 [ ] More than 1,000

Q4. Is your school a member of the Massachusetts Interscholastic Athletic Association?

[ ] Yes [ ] No [ ] Do not know

Q5. Does your school employ an Athletic Trainer?

[ ] Yes [ ] No [ ] Do not know

Q6. Does your school have a concussion management team that includes other school stakeholders (e.g. School Nurse(s), Athletic Trainer(s), Guidance Counselor(s), etc.) that meet to manage return-to-learn and play for students who have experienced a concussion or other head injury?

[ ] Yes [ ] No [ ] Do not know

Q7. Using the scale below, with 0 being *not important at all* and 10 being *very important*, rate how important you believe the sports concussion regulations are to protect the health and safety of the student athletes at your school.

***Not at all important*** 0 1 2 3 4 5 6 7 8 9 10 ***Very important***

Q8. Indicate how often you believe that you are informed in a timely manner when a student athlete experiences a concussion or other head injury that did not occur during extracurricular sports at your school.

[ ] Always [ ] Often [ ] Sometimes [ ] Rarely [ ] Never

Q9. In your experience, what proportion of the following stakeholders are knowledgeable about the requirements of the sports concussion regulations?

|  | Few | Some | Most | All | Do not know |
| --- | --- | --- | --- | --- | --- |
| School Nurses |  |  |  |  |  |
| Teachers |  |  |  |  |  |
| Athletic Directors |  |  |  |  |  |
| Guidance Counselors |  |  |  |  |  |
| School Administrators |  |  |  |  |  |
| Athletic Trainers |  |  |  |  |  |
| Coaches |  |  |  |  |  |
| Students' physicians |  |  |  |  |  |
| Students' parents |  |  |  |  |  |

Q10. Using the scale below, with 0 being ***not at all*** and 10 being ***it has made it impossible to keep up with the demands of my job***, please indicate how much the implementation of the sports concussion regulations has added to your workload.

***Not at all*** 1 2 3 4 5 6 7 8 9 10 ***It has made it impossible to keep up with the demands of my job***

Q11. Baseline ImPACT or comparable neurocognitive testing is provided by your school (either in-person or on-line) for:

[ ] None of the students [ ] All students engaged in any extracurricular athletics at the school

[ ] All students engaged in certain extracurricular athletics at the school. Specify sport(s): ________________________________________________

[ ] All students in certain grades. Specify grade(s): ________________________________________________

[ ] All students in the school.

[ ] Other (please specify): ________________________________________________

Q12. In your experience, how often do you feel that student athletes misrepresent concussion symptoms to return-to-play sooner?

[ ] Always [ ] Often [ ] Sometimes [ ] Rarely [ ] Never

Q13. In your experience, how often do you feel that student athletes misrepresent concussion symptoms to avoid schoolwork (e.g. classes, exams, etc.)?

[ ] Always [ ] Often [ ] Sometimes [ ] Rarely [ ] Never

Q14. The name of your school is:

________________________________________________________________

Q15. Is there anything else you would like to tell us about your experience with the sports concussion regulations?

________________________________________________________________

________________________________________________________________

________________________________________________________________
